# Antioxidant Activities of Extracts and Main Components of Pigeonpea [*Cajanus* *cajan* (L.) Millsp.] Leaves

**DOI:** 10.3390/molecules14031032

**Published:** 2009-03-04

**Authors:** Nan Wu, Kuang Fu, Yu-Jie Fu, Yuan-Gang Zu, Fang-Rong Chang, Yung-Husan Chen, Xiao-Lei Liu, Yu Kong, Wei Liu, Cheng-Bo Gu

**Affiliations:** 1 Key Laboratory of Forest Plant Ecology, Ministry of Education, Northeast Forestry University, Harbin 150040, P.R. China; 2 The Second Affiliated Hospital of Harbin Medical University, Harbin 150086, P.R. China; 3 Graduate Institute of Natural Products, Kaohsiung Medical University, Kaohsiung 807, Taiwan

**Keywords:** *Cajanus cajan* (L.) Millsp., Antioxidant activities, DPPH, β-Carotene.

## Abstract

Antioxidant activities of the aqueous and ethanol extracts of pigeonpea [*Cajanus* *cajan* (L.) Millsp.] leaves, as well as petroleum ether, ethyl acetate, *n*-butanol and water fractions and the four main compounds separated from the ethanol extract, i.e. cajaninstilbene acid (3-hydroxy-4-prenylmethoxystilbene-2-carboxylic acid), pinostrobin, vitexin and orientin, were examined by a DPPH radical-scavenging assay and a β-carotene-linoleic acid test. In the DPPH system, the antioxidant activity of the ethanol extracts was superior to that of the aqueous extracts, with IC_50_ values were 242.01 and 404.91 µg/mL, respectively. Among the four fractions, the ethyl acetate one showed the highest scavenging activity, with an IC_50_ value of 194.98 µg/mL. Cajaninstilbene acid (302.12 µg/mL) and orientin (316.21 µg/mL) showed more efficient radical-scavenging abilities than pinostrobin and vitexin. In the β-carotene-linoleic acid test, the inhibition ratio (%) of the ethyl acetate fraction (94.13%±3.41%) was found to be the highest, being almost equal to the inhibition capacity of the positive control BHT (93.89%±1.45%) at 4 mg/mL. Pinostrobin (>500 µg/mL) and vitexin (>500 µg/mL) showed insignificant antioxidant activities compared with cajaninstilbene (321.53 µg/mL) and orientin (444.61 µg/mL). In general, the ethyl acetate fraction of the ethanol extract showed greater activity than the main compounds in both systems, such results might be attributed to the synergistic effects of the components. The antioxidant activities of all the tested samples were concentration-dependent. Based on the results obtained, we can conclude that the pigeonpea leaf extracts may be valuable natural antioxidant sources and are potentially applicable in both medicine and the healthy food industry.

## Introduction

Pigeonpea [*Cajanus cajan* (L.) Millsp.] is a perennial member of the family leguminosae. Other common names are red gram, Congo pea, Gungo pea, Gunga pea, and no-eye pea. It is an important grain legume crop of rain-field agriculture in the tropics and subtropics. Compared with other grain legumes, pigeonpea ranks only sixth in area and production, but it is used in more diverse ways than others [1,2,3,4]. The extracts or components of pigeonpea are commonly used all over the world for the treatment of diabetes, dysentery, hepatitis and measles, as a febrifuge to stabilize the menstrual period [5,6,7,8]. As a traditional Chinese medicine, the leaves of pigeonpea have been widely used to arrest blood, relieve pain and kill worms [9]. Nowadays, pigeonpea leaves are used for the treatment of wounds, aphtha, bedsores and malaria, as well as diet-induced hypercholesterolemia, etc [10,11,12,13]. Protective effects of extracts from pigeonpea leaf against hypoxic-ischemic brain damage and alcohol-induced liver damage have also been reported [14,15]. Chemical constituent investigations have indicated that pigeonpea leaves are rich in flavonoids and stilbenes, which are considered responsible for the beneficial efficacies of pigeonpea leaves on human health [16,17,18].

Antioxidants are widely used as food additives to provide protection against oxidative degradation of foods by free radicals [19]. Since ancient times, spices added to different types of food to improve flavors are also well known for their antioxidant capacities [20]. In recent decades, there has been great interest in screening essential oils and various plant extracts for natural antioxidants because of their good antioxidant properties. In order to prolong the storage of foods, several synthetic antioxidants such as butylated hydroxytoluene (BHT) and butylated hydroxyanisole (BHA) are used currently, but these substances may be inappropriate for chronic human consumption, as recent publications have mentioned their possible toxic properties for human health and the environment [21,22]. Hence, the development of alternative antioxidants of natural origin has attracted considerable attention and is generally thought to be a desirable development [23].

**Figure 1:**
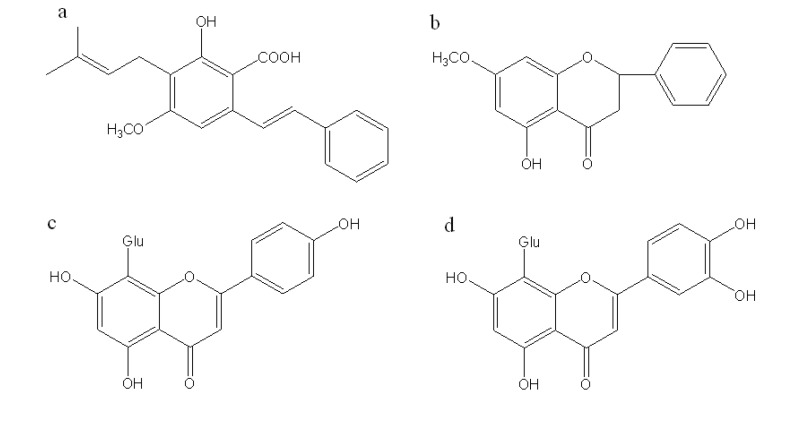
Chemical structures of (a) cajaninstilbene acid, (b) pinostrobin, (c) vitexin and (d) orientin. Glu: glucose.

Though a large number of plants worldwide show strong antioxidant activities [24,25], the antioxidant properties of pigeonpea extracts have not been elucidated before. Therefore, the objective of the present study was to investigate the antioxidant properties of its aqueous and ethanol extracts of pigeonpea leaves, different fractions of the ethanol extract as well as its four main components, namely cajaninstilbene acid, pinostrobin, vitexin and orientin (Figure 1) by DPPH and β-carotene-linoleic acid assays. The present study would offer basic data on the natural antioxidant potential of pigeonpea leaves for the food or pharmaceutical industries, and also provides scientific reference for the large scale usage and exploitation of pigeonpeas as a resource.

## Results and Discussion

### Amounts of total flavonoids and contents of main components

Based on the absorbance values of the various extract solutions, and compared with the standard solutions of rutin equivalents as described below, results obtained in this study revealed that the level of flavonoids in the ethyl acetate fraction of pigeonpea leaves was considerable (Table 1). Aqueous extracts (146.32±3.28 mg/g extract), ethanol extracts (293.45±3.12 mg/g extract), petroleum ether fraction (151.29±1.55 mg/g extract), *n*-butanol fraction (211.37±2.19 mg/g extract) and water fraction (52.43±1.64 mg/g extract) all contained less total flavonoids than the ethyl acetate fraction (384.43±1.58 mg/g extract). Plants often contain substantial amounts of antioxidants, including tocopherols (vitamin E), carotenoids, ascorbic acid, flavonoids and tannins [26]. According to the yields in this experiment, the flavonoid content of the ethanol extracts (293.45±3.12 mg rutin equivalents (RE)/g extract) was equivalent to 57.57 mg RE/gdw, which was higher than that reported for some other leguminosae species with antioxidant activities, such as cassia seed (47.41 ± 2.17 mg RE/gdw), kudzuvine root (2.66 ± 0.05 mg RE/gdw), adzuki bean (5.38 ± 0.15 mg RE/gdw), fermented soybean (1.96 ± 0.10 mg RE/gdw)and white hyacinth bean (2.27 ± 0.09 mg RE/gdw) [27]. 

Table 1 also showed the contents of main components in four fractions of the ethanol extract of pigeonpea leaves. The contents of cajaninstilbene acid and pinostrobin were higher in the petroleum ether fraction, about two-fold that in the ethyl acetate fraction, however, the contents of vitexin and orientin were much less than those in ethyl acetate fraction as well as n-butanol fraction. Overall, the ethyl acetate fraction was found to be rich in all of the four components, with cajaninstilbene acid (37.795±2.13 mg/g extract), pinostrobin (12.339±2.53 mg/g extract), vitexin (21.03±2.23 mg/g extract), and orientin (18.82±1.13 mg/g extract).

**Table 1:** Contents of total flavonoids and main components in the extracts and four fractionsof pigeonpea leaves (*n*=3).

Sample	Contents (mg/g extract)				
	Flavonoids	Cajaninstilbene acid	Pinostrobin	Vitexin	Orientin
Aqueous extracts	146.32±3.28	0.545±0.12	0.89±0.68	1.25±0.21	1.07±0.32
Ethanol extracts	293.45±3.12	32.76±1.67	11.85±1.41	6.21±0.35	6.13±2.21
Petroleum ether fraction	151.29±1.55	69.93±1.39	30.29±2.09	0.253±0.07	0.122±0.09
Ethyl acetate fraction	384.43±1.58	37.795±2.13	12.339±2.53	21.03±2.23	18.82±1.13
*n*-Butanol fraction	211.37±2.19	-	-	5.697±3.26	8.331±1.08
Water fraction	52.43±1.64	-	-	-	4.383±0.24

Values are means ± SD of three determinations. Data in the same column indicate significant difference (p < 0.05).

**Figure 2:**
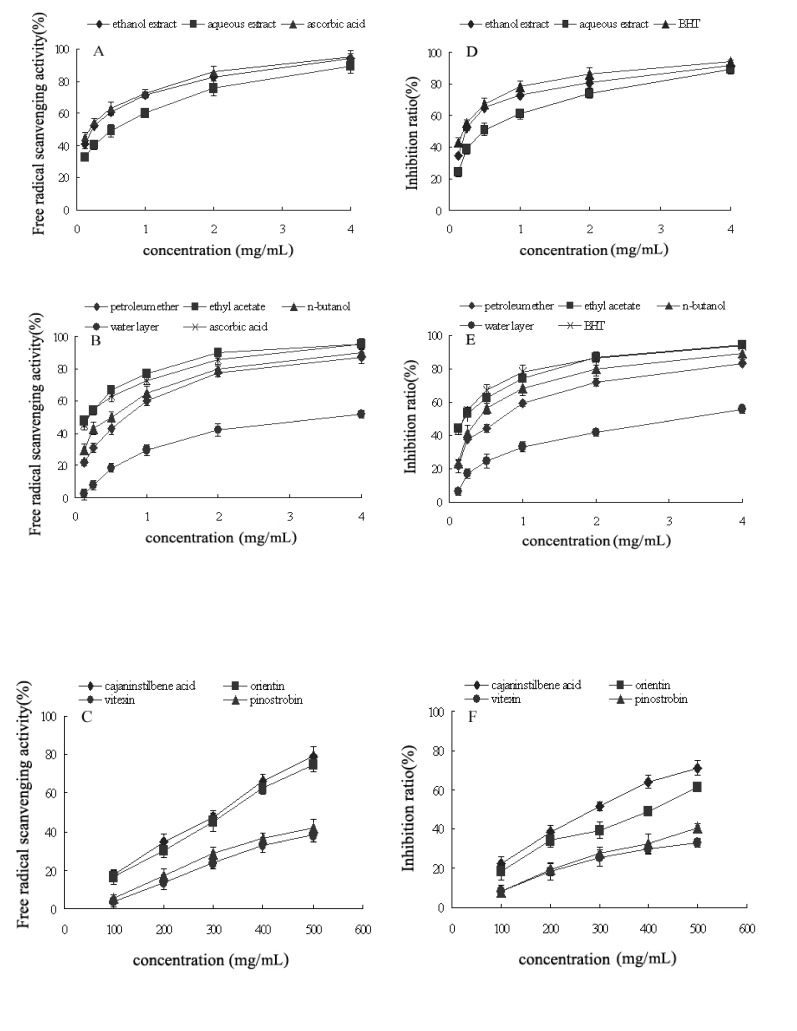
Antioxidant activities of extracts, four fractions and four main components (cajaninstilbene acid, pinostrobin, vitexin and orientin) in pigeonpea leaves (A-C: DPPH radical scavenging assay; D-F: β-carotene-linoleic acid test). Values of each curve are means ± SD (n = 3). P < 0.05.

### Antioxidant activities

Antioxidant activities of the aqueous and ethanol extracts as well as four fractions separated from the ethanol extracts (petroleum ether, ethyl acetate, *n*-butanol, and water fractions) and four main compounds have been determined by two different test systems DPPH and β-carotene-linoleic acid. All of the data are presented in Figure 2 and Table 2. 

In the DPPH test system, free radical-scavenging activity of the ethyl acetate fraction, with an IC_50 _value of 194.98 µg/mL, was superior to those of all tested samples, but lower than that of the positive control ascorbic acid (201.29 µg/mL; p < 0.05). The isolated cajaninstilbene acid and orientin from this extract also exhibited certain antioxidant activity (IC_50_ 302.12 and 316.21µg/mL, respectively). Other samples had IC_50_ values between 242.01 and 2,388.31 µg/mL. 

**Table 2:** IC_50_ values of extracts, four fractions and main components of pigeonpea leaves.

Sample	DPPH radical scavenging assay IC_50_ (µg/mL)	β-carotene-linoleic acid testIC_50_ (µg/mL)
Aqueous extracts	404.91*	475.26†
Ethanol extracts	242.01*	256.88†
Petroleum ether fraction	553.41*	581.99†
Ethyl acetate fraction	194.98*	213.89†
n-Butanol fraction	382.23*	410.47†
Water fraction	2388.31*	2663.91†
Cajaninstilbene acid	302.12*	321.53†
Pinostrobin	>500*	>500†
Vitexin	>500*	>500†
Orientin	316.21*	444.61†
Ascorbic acid	201.29	-
BHT	-	195.74

The symbols * and ^†^indicate very significant difference p < 0.01 with respect to positive control (ascorbic acid or BHT).

In the β-carotene-linoleic acid model system, we could conclude that results were consistent with the data obtained from the DPPH test. The ethanol extracts (91.23% ± 0.42%) showed markedly antioxidant activity, as did the aqueous extracts (89.32% ± 3.28%) at 4 mg/mL. The inhibition ratio of ethyl acetate fraction (94.13% ± 3.41%) was found to be the greatest, and almost equal to the inhibition capacity of the positive control BHT (93.89% ± 1.45%). Vitexin and pinostrobin showed the weakest activity potential in this test system. 

Ganiyu Oboh studied the radical-scavenging abilities of some commonly consumed and underutilized tropical legumes [cowpea (*Vigna unguiculata*), pigeonpea (*Cajanus cajan*) and African yam bean (*Sphenostylis sternocarpa*)] [28]. According to his results, methanol extracts of pigeonpea(brown) showed the highest radical-scavenging ability (54.5%). This value is significantly (*p* *<* 0.05) lower than the free radical scavenging ability of ethanol extracts of pigeonpea in our test. Besides the extract solvent and method, the environmental factors and different species may account for this difference in activity.

Until now, various authors have reported antioxidant activities of plants of the family leguminosae. Some plants showed stronger radical-scavenging abilities than pigeonpea leaves, e.g. methanol and water extracts of *Caesalpinia digyna* root (IC_50_ values are 4.86 ± 0.90 and 89.72 ±1.05), ethanol extract of leaves of *Pseudopiptadenia contorta*, *Platypodium elegans* as well as peanut skins (13.84 ± 0.30 µg/mL, 184.92 ± 2.69 and 30.8 µg/mL, respectively) (*p* *<* 0.05). Meanwhile, more plants showed much weaker radical-scavenging abilities, e.g. methanol extract of fenugreek (*Trigonella foenum graecum*) seeds (350 µg/mL), ethanol extraction of tuberous roots of *Pueraria lobata* (2,482.00 ± 766.11 µg/mL) and *Pueraria mirifica* (2,904.52 ± 30.37 µg/mL) [29,30,31,32,33] (*p* *<* 0.05). From the compare data, pigeonpea leaves had the potential application as natural antioxidant in many fields.

It could be inferred from our results that there was a positive correlation between flavonoid content and antioxidant activity, as the higher activity of the ethyl acetate fraction could be attributed to the higher content of flavonids. The relationship between the chemical structure of flavonoids and their antioxidant activities had been analyzed by Arora *et al*. and van Acker *et al*. [34,35]. According to the results of these studies, a catechol or a pyrogallol type moiety substitution on B ring appeared to be essential for antioxidant activities of flavonoids. The presence of hydroxyl substituents on the flavonoid skeleton boosts activity, whereas methoxyl substitution suppresses the antioxidant activity. The most potent activity of ethyl acetate fraction in our case may be result from compounds with such structures possessing the strongest antioxidant activity. Pinostrobin, vitexin and orientin are all flavonoids, but only orientin showed moderate antioxidant activity in our study. Maybe the lack of hydroxyl substituents was one of the factors contributing to this. Though we didn’t observe antioxidant activities for pinostrobin in our in *vitro* assays, there are reports showing pinostrobin is a potent flavonoid inducer of antioxidant enzymes [36].

From the above results, we also found that cajaninstilbene acid, a member of the stilbenes, showed strong antioxidant activity, equivalent to resveratrol, which was usually considered as a natural antioxidant [37]. The results prompted us to investigate the potential mechanism of its antioxidant activity in the future. Though the IC_50_ value of cajaninstilbene acid was lower than the other three components, the antioxidant activity of petroleum ether fraction which contained abundant cajaninstilbene acid was quite weak. Such results suggest synergistic interactions between flavonoids and stilbenes. In fact, it is very difficult to attribute the antioxidant effect of a total extracts to one or a few active principles, because extracts always contains a mixture of different chemical compounds. Besides the major components, minor components may also make a significant contribution to the antioxidant activity of extracts. Following the results above, we could conclude that the antioxidant activity of extracts of pigeonpea is probably a synergistic effect of their compositions. 

In conclusion, results in the present study could be an effective introduction to the antioxidant activities of pigeonpea leaves, and provided evidence that ethyl acetate fraction of the ethanol extracts of pigeonpea leaves may provide potential natural antioxidants for the food industry and other fields. However, further studies are urgently needed for screening for the active components with enhanced antioxidant activity properties in pigeonpea leaves.

## Experimental

### General

Ethanol, methanol, chloroform, petroleum ether, ethyl acetate, *n*-butanol of analytical grade were obtained from Beijing Chemical Reagents Co. (Beijing, P.R. China). 2,2-Diphenyl-1-picrylhydrazyl (DPPH), β-carotene, BHT and ascorbic acid (VC) were purchased from Sigma-Aldrich (Steinheim, Germany). The water used in all experiments was double-distilled. CSA (≥98%, HPLC grade), pinostrobin (≥95%, HPLC grade), vitexin (≥98%, HPLC grade) and orientin (≥98%, HPLC grade) were separated and purified in the Key Laboratory of Forest Plant Ecology. The structures were confirmed by comparing the IR, ^1^H- and ^13^C-nuclear magnetic resonance (NMR) and MS data with the reported data [11,38,39]. 

### Plant material

The dried pigeonpea leaves were collected from Hainan Province, P.R. China, and authenticated by Profossor Shao-Quan Nie from the Key Laboratory of Forest Plant Ecology, Ministry of Education, Northeast Forestry University, P.R. China. Voucher specimens were deposited in the herbarium of this Key Laboratory. The pigeonpea leaves were pulverized to 40 mesh, then stored in a dry place at room temperature until use. 

### Preparation of the ethanol and aqueous extracts

The ethanol extracts were prepared by extraction under reflux: pigeonpea leaves (50 g) were added to 80% ethanol (400 mL) and extracted at 80 °C for 1 h. The ethanol was evaporated under vacuum to obtain the residue (yield: 12.29% w/w). The residue was stored at 4 °C until used. The aqueous extracts of pigeonpea leaves were prepared as follows: pigeonpea leaves (50 g) were added to boiling distilled water (400 mL) and extracted under reflux for 1 h at 80 °C, then filtered and freeze-dried. The powder (yield: 8.6% w/w) was stored at 4 °C until use. 

The dried 80% ethanol extract residue was suspended in distilled water (100 mL) and the resulting aqueous suspension was partitioned sequentially with petroleum ether, ethyl acetate and *n*-butanol in a 1:1(v/v) ratio three times at room temperature. The resulting three extracts and remaining water phase were evaporated under vacuum to dryness to give the corresponding petroleum ether, ethyl acetate, *n*-butanol, and water fraction powders. These powders were kept at 4 °C in the dark until further analysis.

### Separation of target components

The ethanol extract of pigeonpea leaves was subjected to silica gel column chromatography eluting with petroleum ether-chloroform and methanol. The petroleum ether-chloroform eluted fractions contained pinostrobin and cajaninstilbene acid, chloroform-methanol eluted fractions contained orientin and vitexin. The two eluted fractions were further rechromatographed on middle-pressure silica gel columns, with petroleum ether-chloroform and chloroform-methanol gradients, respectively, and then purified by crystallization and recrystallization to obtain pinostrobin (180.00 mg), cajaninstilbene acid (230.60 mg), vitexin (19.30 mg) and orientin (15.80 mg). Their identities were confirmed by IR, ^1^H- and ^13^C-nuclear magnetic resonance (NMR) and MS analysis. 

### HPLC analysis

HPLC analyses were performed on a Waters high performance liquid chromatography system (Waters Corporation, USA) equipped with Model Delta 600 pump, 2996 photodiode array detector and Millennium32 system software. The column was a HIQ Sil C18V reversed-phase column (250 mm×4.6 mm I.D., Kya Tech, Hachioji City, Japan); the mobile phase was a mixture of solvent A (methanol) and B (0.1% formic acid in water, v/v), according to a linear gradient, lasting 65 min, changing from 90% A to 33% A in 20 min, at a flow rate of 1.0 mL/min. The signals at wavelengths of 259.2 nm (cajaninstilbene acid), 290 nm (pinostrobin), 330 nm (vitexin) and 345 nm (orientin) were stored and collected. 

### Determination of total flavonoids

The flavonoids contents in the extracts were determined spectrophotometrically using the method of Ordon-Ez *et al*. based on the formation of a flavonoid-aluminum complex [40]. An amount of 2% ethanolic AlCl_3_ solution (0.5 mL) of was added to 0.5 mL of sample. After 1 h at room temperature, the absorbance was measured at 420 nm. A yellow color indicated the presence of flavonoids. Extract samples were evaluated at a final concentration of 0.1 mg/mL. Total flavonoids contents were calculated as rutin (mg/g extract).

### Determination of antioxidant activity

*DPPH radical scavenging assay:* The hydrogen atom or electron donation abilities of the extracts, along with the four fractions separated from the ethanol extracts (petroleum ether fraction, ethyl acetate fraction, *n*-butanol fraction and water fraction) and the pure compounds were measured from the bleaching of a purple-colored ethanol solution of DPPH. This spectrophotometric assay uses the stable radical 2,2-diphenyl-1-picrylhydrazyl (DPPH) as a reagent [41]. An aliquot of the sample (100 µL) was mixed with ethanol (1.4 mL) and then added to 0.004% DPPH (1 mL, Sigma-Aldrich) in ethanol. The mixture was shaken vigorously and then immediately placed in a UV-Vis spectrophotometer (UNICO, Shanghai, China) to monitor the decrease in absorbance at 517 nm. Monitoring was continued for 70 min until the reaction reached a plateau. Ascorbic acid (Sigma-Aldrich), a stable antioxidant, was used as a synthetic reference. The radical-scavenging activities of samples, expressed as percentage inhibition of DPPH, were calculated according to the formula: Inhibition percentage (Ip) = [(AB-AA)/AB]×100 [42] where AB and AA are the absorbance values of the blank sample and of the tested samples checked after 70 min, respectively.

*β-carotene-linoleic acid test:* Antioxidant activity of the samples was determined using the β-carotene-linoleic acid test [43]. Approximately 10 mg of β-carotene (type I synthetic, Sigma–Aldrich) was dissolved in chloroform (10 mL). The carotene–chloroform solution, (0.2 mL) was pipetted into a boiling flask containing linoleic acid (20 mg, Sigma–Aldrich) and 200 mg Tween^®^ 40 (Sigma–Aldrich). Chloroform was removed using a rotary evaporator (RE-52AA) at 40 °C for 5 min, and distilled water (50 mL) was added to the residue slowly with vigorous agitation, to form an emulsion. A portion of the emulsion (5 mL) was added to a tube containing the sample solution (0.2 mL) and the absorbance was immediately measured at 470 nm against a blank, consisting of an emulsion without β-carotene. The tubes were placed in a water bath at 50 °C and the oxidation of the emulsion was monitored spectrophoto-metrically by measuring absorbance at 470 nm over a 60 min period. Control samples contained 200 µL of water instead. Butylated hydroxytoluene (BHT, Sigma–Aldrich), a stable antioxidant, was used as a synthetic reference. The antioxidant activity was expressed as inhibition percentage with reference to the control after a 60 min incubation using the following equation: AA = 100(DR_C_ -DR_S_)/DR_C_, where AA = antioxidant activity; DR_C_ = degradation rate of the control = [ln(a/b)/60]; DR_S_ = degradation rate in presence of the sample = [ln(a/b)/60]; a = absorbance at time 0; b = absorbance at 60 min.

### Statistical analysis

Results of the research were tested for statistical significance by one-way ANOVA. Differences were considered statistically significant at the P < 0.05 level. 

## References

[1] Chakraborty S.K., Kumbhar. B.K., Sarkar B.C. (2007). Process parameter optimization for instant pigeonpea dhal using response surface methodology. J. Food. Eng..

[2] Salunkhe D.K., Chavan J.K., Kadam S.S. (1986). Pigeonpea as an important food source. Crit. Rev. Food Sci..

[3] Fu Y.J., Zu Y.G., Liu W., Efferth T., Zhang N.J., Liu X.N., Kong Y. (2006). Optimization of luteolin separation from pigeonpea [*Cajanus cajan* (L.) Millsp.] leaves by macroporous resins. J. Chromatogr. A.

[4] Fu Y.J., Zu Y.G., Liu W., Hou C.L., Chen L.Y., Li S.M., Shi X.G., Tong M.H. (1139). Preparative separation of vitexin and isovitexin from pigeonpea extracts with macroporous resins. J. Chromatogr. A.

[5] Amalraj T., Ignacimuthu S. (1998). Evaluation of the hypoglycaemic effect of *Cajanus cajan* (seeds) in mice. Indian J. Exp. Biol..

[6] Grover J.K., Yadav S., Vats V.J. (2002). Medicinal plants of India with anti-diabetic potential. J. Ethnopharmacol..

[7] Duke J.A., Vasquez R. (1994). Amazonian Ethnobotanical Dictionary.

[8] Abbiw D.K. (1990). Useful Plants of Ghana.

[9] Tang Y., Wang B., Zhou X.J. (1999). Effect of external application of herbal cajani preparation on the fibronection content during healing process of open wound. J. Guangzhou U. Tradit. Chin. Med..

[10] Aiyeloja A.A., Bello O.A. (2006). Ethnobotanical potentials of common herbs in Nigeria: A case study of Enugu state. Educat. Res. Rev..

[11] Chen D.H., Li H.Y, Lin H. (1985). Studies on chemical constituents in pigeonpea leaves. Chin. Tradit. Herb Drugs.

[12] Li Z.H., Zhou C.H., Zhang J.Y. (2001). The present status of study and utilization of pigeonpea in China and its prospects. Forest Res..

[13] Luo Q.F., Sun L., Si J.Y., Chen D.H. (2008). Hypocholesterolemic effect of stilbenes containing extract-fraction from *Cajanus cajan* L. on diet-induced hypercholesterolemia in mice. Phytomedicine.

[14] Huang G.Y., Liao X.Z., Liao H.F., Deng S.J., Tan Y.H., Zhou J.Y. (2006). Studies on water-soluble extracts from *Cajanus cajan* leaf against hypoxic-ischemic brain damage. Tradit. Chin. Drug Res. Clin. Pharmacol.

[15] Kundu R., Dasgupta S., Biswas A., Bhattacharya A., Pal B.C., Bandyopadhyay D., Bhattacharya S., Bhattacharya S. (2008). Cajanus cajan Linn. (Leguminosae) prevents alcohol-induced rat liver damage and augments cytoprotective function. J. Ethnopharmacol..

[16] Duker-Eshun G., Jaroszewski J.W, Asomaning W.A., Oppong-Boachie F, Christensen S.B. (2004). Antiplasmodial constituents of *Cajanus cajan*. Phytother Res..

[17] Zu Y.G., Fu Y.J., Liu W., Hou C.L., Kong Y. (2006). Simultaneous determination of four flavonoids in pigeonpea [*Cajanus cajan* (L.) Millsp.] leaves using RP-LC-DAD. Chromatographia.

[18] Zheng Y.Y., Yang J., Chen D.H., Sun L. (2007). Effects of the stilbene extracts from *Cajanus cajan* L. on ovariectomy-induced bone loss in rats. Acta Pharm. Sin..

[19] Gülçin İ., Oktay M., Küfrevioğlu Ö. İ., Aslan A. (2002). Determination of antioxidant activity of lichen *Cetraria islandica* (L). Ach. J. Ethnopharmacol..

[20] Madsen H.L., Bertelsen G. (1995). Spices as antioxidants. Trends Food Sci. Tech..

[21] Ito N., Hirose M., Fukushima H., Tsuda T., Shirai T., Tatenatsu M. (1986). Studies on antioxidants: Their carcinogenic and modifying effects on chemical carcinogens. Food Chem. Toxicol..

[22] Stich H.F. (1991). The beneficial and hazardous effects of simple phenolic compounds. Mutat. Res..

[23] Jia Z.B, Tao F., Guo L., Tao G.J., Ding X.L. (2007). Antioxidant properties of extracts from juemingzi (*Cassia tora* L.) evaluated in vitro. LWT.

[24] Baratto M.C., Tattini M., Galardi C., Pinelli P., Romani A., Visiolid F., Basosi R., Poqni R. (2003). Antioxidant activity of Galloyl quinic derivatives isolated from *Pistacia lentiscus* leaves. Free Radical Res..

[25] Katalynic V., Milos M., Kulisic T., Jukic M. (2006). Screening of 70 medicinal plant extracts for antioxidant capacity and total phenols. Food Chem..

[26] Larson R.A. (1988). The antioxidants of higher plants. Phytochemistry.

[27] Liu H.Y., Qiu N.X., Ding H.H., Yao R.Q. (2008). Polyphenols contents and antioxidant capacity of 68 Chinese herbals suitable for medical or food uses. Food Res. Int..

[28] Oboh G. (2006). Antioxidant properties of some commonly consumed and underutilized tropical legumes. Eur. Food Res. Technol..

[29] Srinivasan R., Chandrasekar M.J.N., Nanjan M.J., Suresh B. (2007). Antioxidant activity of *Caesalpinia digyna* root. J. Ethnopharmacol..

[30] Mensor L.L., Menezes F.S., Leitão G.G., Reis A.S., dos Santos T.C., Coube C.S., Leitão S.G. (2001). Screening of Brazilian plant extracts for antioxidant activity by the use of DPPH free radical method. Phytother. Res..

[31] Wang J., Yuan X.P., Jin Z.Y., Tian Y., Song H.L. (2007). Free radical and reactive oxygen species scavenging activities of peanut skins extract. Food Chem..

[32] Kaviarasan S., Naik G.H., Gangabhagirathi R., Anuradha C.V., Priyadarsini K.I. (2007). In vitro studies on antiradical and antioxidant activities of fenugreek (*Trigonella foenum graecum*) seeds. Food Chem..

[33] Cherdshewasart W., Sutjit W. (2008). Correlation of antioxidant activity and major isoflavonoid contents of the phytoestrogen-rich *Pueraria mirifica* and *Pueraria lobata* tubers. Phytomedicine.

[34] Arora A., Nair M.G., Strasburg G.M. (1998). Structure-activity relationships for antioxidant activities of a series of flavonoids in a liposomal system. Free Radical Biol. Med..

[35] Van Acker S.A.B.E., Van den Berg D.J., Tromp M.N.J.L., Griffioen D.H., Van Bennekom W.P., Van der Vijgh W.J., Bast A. (1996). Structural aspects of antioxidant activity of flavonoids. Free Radical Biol. Med..

[36] Jed W., Fahey, Katherine K. (2002). Stephenson Pinostrobin from Honey and Thai Ginger (*Boesenbergia pandurata*): A Potent Flavonoid Inducer of Mammalian Phase 2 Chemoprotective and Antioxidant Enzymes.. J. Agric. Food Chem..

[37] Iacopini P., Baldi M., Storchi P., Sebastiani L. (2008). Catechin, epicatechin, quercetin, rutin and resveratrol in red grape: Content, in vitro antioxidant activity and interactions. J. Food Compos. Anal..

[38] Cooksey C.J., Dahiya J.S., Garratt P.J., Strange R.N. (1982). Two novel stilbene-2-carboxylic acid phytoalexins from *Cajanus cajan*. Phytochemistry.

[39] Ordoñez A.A.L., Gomez J.D., Vattuone M.A., Isla M.I. (2006). Antioxidant activities of *Sechium edule* (Jacq.) Swart extracts. Food Chem..

[40] Zhou X., Peng J., Fan G.., Wu Y. (2005). Isolation and purification of flavonoid glycosides from *Trollius lebebouri* using high-speed counter-counter chromatography by stepwise increasing the flow-rate of the mobile phase. J. Chromatogr. A.

[41] Amarowicz R., Pegg R.B., Moghaddam P.R., Barl B., Weil J. (2004). A Free-radical scavenging capacity and antioxidant activity of selected plant species from Canadian prairies. Food Chem..

[42] Yen G.C., Duh P.D. (1994). Scavenging effect of methanolic extracts of peanut hulls on free-radical and active-oxygen species. J. Agr. Food Chem..

[43] Taga M.S., Miller E.E., Pratt D.E. (1984). Chia seeds as a source of natural lipid antioxidant. J. Am. Oil Chem. Soc..

